# Acquired thrombotic thrombocytopenic purpura after first vaccination dose of BNT162b2 mRNA COVID-19 vaccine

**DOI:** 10.1007/s00277-021-04584-y

**Published:** 2021-07-26

**Authors:** Johannes Ruhe, Ulf Schnetzke, Karim Kentouche, Florian Prims, Michael Baier, Konstantin Herfurth, Mandy Schlosser, Martin Busch, Andreas Hochhaus, Gunter Wolf

**Affiliations:** 1 Klinik Für Innere Medizin III, Abteilung Für Nephrologie, Universitätsklinikum Jena, Jena, Germany; 2 Klinik Für Innere Medizin II, Abteilung Für Hämatologie Und Internistische Onkologie, Universitätsklinikum Jena, Jena, Germany; 3 Klinik Für Kinder- Und Jugendmedizin, Abteilung Für Hämatologie Und Onkologie, Universitätsklinikum Jena, Jena, Germany; 4 Klinik Für Innere Medizin, Abteilung Hämatologie Und Onkologie, SRH Klinikum Burgenlandkreis Naumburg, Naumburg, Germany; 5 Institut Für Medizinische Mikrobiologie, Universitätsklinikum Jena, Jena, Germany

Dear Editor,

Thrombotic thrombocytopenic purpura (TTP) may occur after vaccinations [1–3]. Here, we report a case of severe TTP early after vaccination against COVID-19.

An 84-year-old female patient was admitted to the hospital with partial hemiplegia, scattered petechiae, and severe arterial hypertension. Cerebral magnetic resonance imaging (MRI) revealed multiple subacute emboli without vessel occlusion. Laboratory findings showed thrombocytopenia (45 × 10^9^/l), Coombs negative hemolytic anemia (hemoglobin 7.9 g/dl; schistocytes 42‰, haptoglobin < 10 mg/dl; total serum bilirubin 2455 mg/dl; Fig. 1), and acute renal failure (serum creatinine 1.95 mg/dl).

**Fig. 1:**
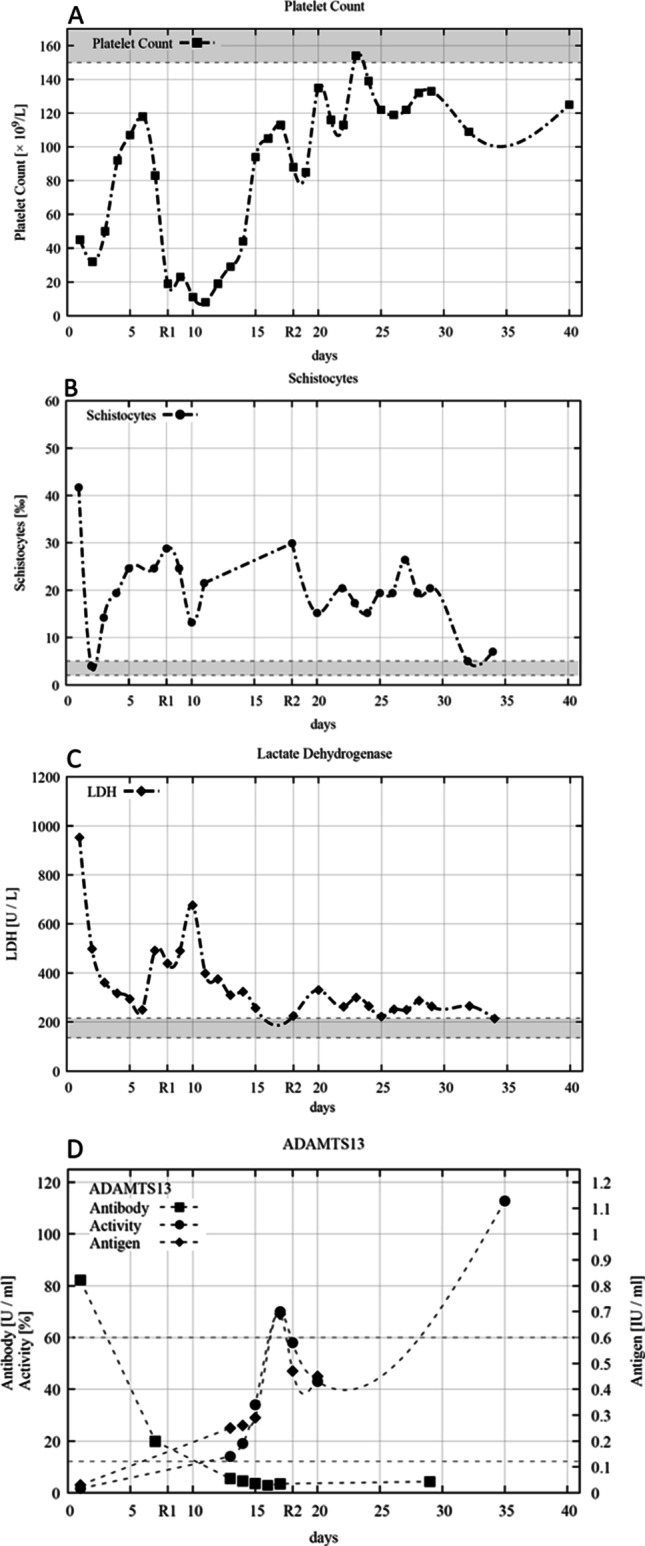
Platelet counts (**A**), schistocytes on a peripheral blood smear (**B**), lactate dehydrogenase (LDH) (**C**), and inhibitory ADAMTS13-antibody, ADAMTS13-activity, and ADAMTS13-antigen (**D**) during the initial 40-day follow-up. Plasma exchange was performed daily until day 18 except for day 9. Rituximab (1000 mg) was applied on days 8 (R1) and 18 (R2). Normal value ranges are marked in gray

Sixteen days before admission, the patient received the first vaccination dose of BNT162b2 (Comirnaty®; Biontech/Pfizer) against COVID-19. Anti-platelet factor 4–IgG was 0.04 U/ml (normal range < 1.0 U/ml); HIPA (heparin-induced platelet antibody) and PIPA (platelet-iodinated protein A) tests were negative at admission so that a SARS-CoV-2 vaccine-induced immune TTP was rather unlikely [4]. Vaccination-related IgG-antibodies against the spike protein were detected (28.6 AU/ml; normal range < 12 AU/ml), without evidence of active or past SARS-CoV-2 infection (negative nucleocapside-IgG and SARS-CoV-2-PCR). Suspecting an acquired TTP, corticosteroid, and plasma exchange therapy (PEX) with fresh frozen plasma were initiated. TTP could be confirmed with an ADAMTS13 activity of 1.6% (60–121%), ADAMTS13-antigen 0.03 IU/ml (0.41–1.41 IU/ml), and inhibitory ADAMTS13-antibodies of 82.2 U/ml (< 12 U/ml). The platelet count increased to 118 × 10^9^/l at day 6 of daily PEX.

After an acute transient loss of consciousness and a sudden drop in platelet count (19 × 10^9^/l) at day 8, 1000 mg rituximab (RTX) was applied in addition to a second corticosteroid pulse. ADAMTS13 antibodies were reduced but still positive (19.9 U/ml); ADAMTS13 activity was 14%. To prevent RTX washout, daily PEX was interrupted for 36 h. As hemolysis may have been aggravated by severe arterial hypertension, likely mediated by TTP-associated endothelial activation stimulating the renin-angiotensin-system, we ensured the consequent administration of angiotensin receptor blocker (candesartan). In the following days, the platelet count and clinical condition improved. After 17 sessions of PEX and the second administration of 1000 mg RTX (day 18), a partial remission was reached with platelets constantly above 100 × 10^9^/l, stabilized red blood cell count (hemoglobin > 9 g/dl), normalized kidney function (creatinine 0.6 mg/dl), and strongly regredient neurologic symptoms. Furthermore, a sufficient proof of ADAMTS13 activity (43–70%) and normalized ADAMTS13 antibodies, lactate dehydrogenase, and other hemolytic parameters supported the findings of clinical improvement (Fig. 1). However, schistocytes were still increased, suggesting possible ongoing mechanical hemolytic activity. Initially increased SARS-CoV-2 IgG and anti-spike titer were normalized and non-detectable at days 9 and 18 as a consequence of daily PEX and the use of rituximab. It can be assumed that no sufficient protection by vaccination could be achieved.

TTP has been described as a complication in COVID-19 patients [5]. A mechanism might be excessive von Willebrand factor (vWF) liberation from the endothelium, exceeding ADAMTS13 capacity for cleaving [6, 7]. Furthermore, Sissa et al. reported about a relapse of TTP 6 days after the second dose administration of BNT162b2 [8]. So far, precise immunological mechanisms remain unclear, but associations to vaccinations as a potential immunological trigger for the formation of antibodies against ADAMTS13 have been published earlier [1–3]. A pre-vaccination gene expression pattern might be an explanation for developing autoantibodies following vaccination [9].

To our knowledge, this is the first case of a primary manifestation of acquired TTP associated with vaccination with BNT162b2 especially in an older woman who is otherwise not particularly prone to having TTP. TTP should be considered in patients with thrombocytopenia after vaccination against COVID-19 and be added to the safety profile of BNT162b2 [10].

## References

[1] Dias PJ, Gopal S (2009). Refractory thrombotic thrombocytopenic purpura following influenza vaccination. Anaesthesia.

[2] Kojima Y, Ohashi H, Nakamura T (2014). Acute thrombotic thrombocytopenic purpura after pneumococcal vaccination. Blood Coagul Fibrinolysis.

[3] Hermann R, Pfeil A, Busch M (2010). Schwerste thrombotisch-thrombozytopenische Purpura (TTP) nach H1N1-Vakzinierung. Med Klin.

[4] Greinacher A, Thiele T, Warkentin TE et al (2021) Thrombotic thrombocytopenia after ChAdOx1 nCov-19 vaccination. New Eng J Med. 10.1056/NEJMoa2104840 10.1056/NEJMoa2104840 PMC8095372 33835769

[5] Tehrani HA, Darnahal M, Vaezi M (2021). COVID-19 associated thrombotic thrombocytopenic purpura (TTP) ; a case series and mini-review. Int immunopharmacol.

[6] Doevelaar AAN, Bachmann M, Hölzer B (2021). von Willebrand factor multimer formation contributes to immunothrombosis in coronavirus disease. Crit Care Med.

[7] Arulkumaran N, Thomas M, Brealey D (2020). Plasma exchange for COVID-19 thrombo-inflammatory disease. EJHaem.

[8] Sissa, C, Al-Khaffaf A, Frattini F et al (2021) Relapse of thrombotic thrombocytopenic purpura after COVID-19 vaccine. Transfus Apher Sci. 10.1016/j.transci.2021.103145 10.1016/j.transci.2021.103145 PMC8051010 33888416

[9] Sobolev O, Binda E, O’Farrell S (2016). Adjuvanted influenza-H1N1 vaccination reveals lymphoid signatures of age-dependent early responses and of clinical adverse events. Nat immunol.

[10] Polack FP, Thomas SJ, Kitchin N (2020). Safety and efficacy of the BNT162b2 mRNA Covid-19 vaccine. N Engl J Med.

